# Optimized protocol for culturing and extracting DNA from fungal isolates associated with brown spot needle blight in pine trees

**DOI:** 10.1371/journal.pone.0337218

**Published:** 2025-11-19

**Authors:** Temitope Ruth Folorunso, Gabriel Silva, Marilis E. Girón, Tess Lindow, Micah Persyn, Lori Eckhardt, Janna R. Willoughby

**Affiliations:** 1 College of Forestry, Wildlife, and Environment, Auburn University, Auburn, Alabama, United States of America; 2 Department of Agricultural Sciences, F, Municipio de San Antonio de Oriente, Francisco Morazán, Tegucigalpa, Honduras; ICAR-National Rice Research Institute, INDIA

## Abstract

Effective culturing and DNA extraction protocols are essential for advancing research on fungal pathogens of brown spot needle blight (BSNB) that infect loblolly pine (*Pinus taeda*) and other *Pinus* species. We evaluated the performance of four widely used fungal media, including Sabouraud dextrose, malt extract, potato dextrose, and yeast extract peptone dextrose, in both solid (agar) and liquid (broth) formats, quantifying fungal growth through colony diameter and biomass accumulation over a three-week period. Sabouraud dextrose agar and broth consistently supported the most rapid and extensive growth in both formats, while potato dextrose underperformed across these metrics. To identify an optimal protocol for downstream molecular applications, we also compared four DNA extraction methods, three of which were modified variants of the CTAB (cetyl-trimethyl-ammonium bromide) chemistry as well as the Qiagen DNeasy kit following the yeast DNA extraction protocol. DNA yield, quantified by fluorometry, was highest for the high-salt CTAB polyvinylpyrrolidone (PVP) protocol and DNA purity (assessed by 260/280 absorbance ratio) was optimal for both PVP and Qiagen extractions. From these comparisons, we suggest that Sabouraud dextrose culturing combined with CTAB PVP extraction for use as a robust and accessible pipeline for generating high-quality fungal DNA.

## Introduction

**Fig 1:**
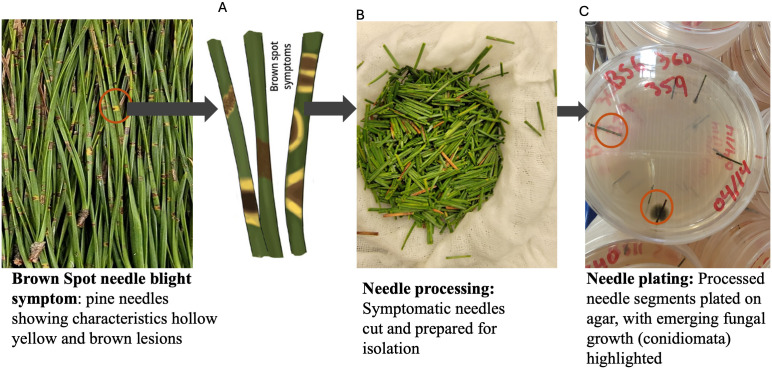
Workflow for fungal isolation from symptomatic pine needles. Symptomatic loblolly pine needles showing characteristics of hollow yellow and brown spot lesions (A) were collected from the field, processes by cutting into small segments for isolation **(B)**, and subsequently plated on agar medium **(C)**, where fungal growth and conidiomata development were observed.

Brown Spot Needle Blight (BSNB) is a fungal disease that affects at least 53 *Pinus* species worldwide [1]. Concern over BSNB has increased due to the occurrence of the disease in new hosts, including loblolly pine (*Pinus taeda*), which supports a large timber industry in many locations [2]. The primary causal agent of this disease is *Lecanosticta acicola* (Thumen H. Sydow), formerly known as *Mycosphaerella dearnessii* [3], although other pathogenic fungi are also likely involved in this complex disease system. For example, endophytic fungi such as *Sydowia polyspora* and *Diplodia sapinea* can produce BSNB symptoms [4,5]. BSNB infection causes the girdling of pine needles and initially presents as yellow-brown bands that develop into multiple infection spots (Fig 1) that tend to result in significant defoliation and, eventually, tree mortality [6,7]. Although symptoms usually appear during the growing season [8], infective spores can be spread by wind-dispersed ascospores and conidiospores year-round [9]. This disease is particularly concerning in the southeastern U.S. because of the multi-billion-dollar pine timber industry that operates in the region [10,11]. Damage to these pine timber stands caused by BSNB infection results in significant economic damage to the forestry industry [1,12].

Effective control of BSNB is hindered by the diversity of *Pinus* hosts and by fundamental gaps in our understanding of the pathogen’s biology. Current management tactics, including prescribed fire and fungicide applications, can lower inoculum loads, yet neither offers a durable, species-wide solution [13,14]. For instance, a carefully timed burn can suppress BSNB on longleaf pine (*Pinus* *palustris*) seedlings, but the same treatment is impossible for loblolly pine (*P. taeda*), whose seedlings are fire-intolerant. Consequently, new management approaches are needed, and their development will, at least partially, depend on deeper insight into the genetic and evolutionary potential of BSNB causing fungi [15]. Although mycologists have tested a variety of culture media to isolate BSNB fungal agents [16], many prove unsuitable for complex downstream processing because these fungi complete their life cycle primarily on pine needles rather than in soil or wood [17]. Selecting a medium that supports reliable culturing is critical for downstream research, particularly when large quantities of high molecular weight fungal DNA is required.

Molecular studies are increasingly used to track pathogen movement and examine resistance characteristics in host species [18,19]. These approaches rely on high-quality DNA, making the optimization of DNA extraction protocols from fungal cultures a critical foundation for future research and management. While commercial kits offer convenience and efficiency, certain fungal pathogens require chemically intensive methods. For instance, cetyltrimethylammonium bromide (CTAB) has been widely used to extract DNA from tissues rich in phenolic compounds [20]. However, despite its widespread application, CTAB-based protocols do not always yield high molecular weight DNA in some organisms [21].

Fungal DNA extraction protocols are often optimized for yeast or clinical isolates, with few tailored solutions for forest pathogens. In this project, we systematically and quantitatively compared commonly used fungal growth media and DNA extraction protocols to identify methods that reliably produce sufficient mycelial biomass and high-molecular-weight DNA. These optimized combinations provide a robust foundation for downstream applications such as long-read sequencing. To do so, we evaluated growth rate across culture media types and compared DNA extraction protocols based on high-molecular weight DNA yield. By identifying a high-yield, reproducible protocol that supports both biomass accumulation and DNA integrity, this work addresses a major obstacle in fungal genomics workflows and provides a practical template for adaptation to other fungal systems. Importantly, this study delivers targeted protocol for BSNB pathogens, filling a critical gap by offering standardized benchmarks that accelerate ongoing genomic and molecular research on this emerging forest disease.

## Materials and methods

### Sample collection

We sampled infected loblolly pine trees with a symptom of brown spot needle blight disease as in Fig 1A from the field across Alabama counties and subsequently used in this study. Needle samples were processed within three days of collection in the Forest Health Laboratory at Auburn University. We obtained five isolates from loblolly pine and one from longleaf pine. These six isolates were evaluated, each in triplicate, across three weekly time points, yielding 72 broth samples and 72 solid-media samples. For DNA extraction, broth samples were processed in three replicates across four extraction protocols, totaling 18 samples per media type and 256 extractions overall.

### Fungal culturing

We considered four media types and their suitability to generating sufficient fungal biomass to support downstream DNA extraction and sequencing, including potato dextrose (PD; [22]); yeast extract peptone dextrose (YEPD; [23]), Sabouraud dextrose (SD; [24]) and 3% malt extract (ME; [25]). For all four media, we considered both solid and broth preparations, and media was prepared following the same protocol except for the absence of agar in the broth media (Table 1). Following dissolution of all components, each medium was sterilized by autoclaving for 20 minutes at 121°C, then poured into sterile petri dishes (solid media, 100 x 15 mm) or Erlenmeyer flasks (broth media) under a laminar flow hood. Media were incubated for ~42h at 22 °C to confirm no contamination [26].

**Table 1:** Composition of fungal culture media used for growth assays.

Reagents	Potato dextrose	Yeast extract peptone dextrose	Sabouraud dextrose	Malt extract
Potato extract	4	—	—	—
Malt extract	—	—	—	30
Dextrose	20	20	40	—
Peptone	—	20	10	5
Yeast Extract	—	10	—	—
Agar (solid media)	15	15	15	15

This table shows the grams of each reagent used to prepare 1L of media. Note that agar was added only to solid media.

For all culture media, we used 4–5 symptomatic needles, those possessing brown spots indicative of BSNB, cut into 3–4 cm pieces long (Fig 1B). These needle pieces were surface sterilized using bleach solution (7.5% of sodium hypochlorite) for 30 seconds, rinsed with distilled water, washed with 70% ethanol for 30 seconds, and then rinsed again with distilled water [27]. Sterilized needle pieces were plated radially in four replicates (Fig 1C). Each fungal isolate was cultured on the four different types of media (Table 1). Fungal growth was measured over a period of 3 weeks, with weekly measurements of colony diameter and observations of morphological features such as pigmentation and sporulation in solid media. For broth cultures, fungi biomass was measured by pelleting the mycelia through centrifugation. Biomass was harvested separately for each replicate and media type, then weighed at the end of each week over a three-week period. The average biomass per sample for each media condition calculated from replicates were recorded and used for the growth rate analysis.

To evaluate the effect of media type on fungal growth, we conducted separate analyses for two response variables: growth rate and total growth, using data collected from both solid and liquid media experiments. For each dataset, growth rate was calculated as the change in colony diameter (solid media) or biomass (liquid media) between consecutive time points, divided by the elapsed time in weeks. Total growth was defined as the final recorded value after three weeks of incubation (colony diameter for solid media and wet biomass for liquid media). In separate models for both growth rate and total growth, we used linear models (lm) in R, version 4.1.0 [28] with media type as a categorical predictor. We also ran separate models for solid and broth media as these growth measurement approaches were substantially different between these culture methods. All models were specified without intercepts to directly estimate the mean response for each media type. Estimated marginal means and 95% confidence intervals for each media type were computed using the emmeans package [29].

### DNA extraction and amplification

To support comparison of DNA extraction methods, we subcultured mycelia from the culture comparisons onto smaller petri dishes (60 x 15 mm) to obtain pure fungal colonies. After one additional week of growth, the pure colony was incubated for a period of 2 weeks in liquid media, after which the harvested mycelia were transferred to 50 mL Falcon tubes and centrifuged at 5,000 × g for 5 minutes. The resulting fungal pellets were then used to evaluate four extraction workflows (Table 2) that were built on a common CTAB (cetyl-trimethyl-ammonium bromide) organic-solvent core. The first protocol combined a 3% CTAB with 1% high-salt SDS (sodium dodecyl sulfate) cleanup to remove phenolics and residual polysaccharides, aimed at enhancing lysis of heavily melanized hyphae. The second protocol incorporated PVP-10 (polyvinylpyrrolidone; 10 g L ⁻ ¹) clean-up to the 1% high-salt 3% CTAB, left overnight for maximum precipitation of DNA using isopropanol and ammonium acetate. The third protocol was a standard 2% CTAB extraction buffer mixed and 1% SDS [20]. Finally, we considered the Qiagen DNeasy Blood and tissue kit (yeast extraction protocol) used according to the manufacturer’s instructions to serve as the commercial kit comparison (Table 2). All methods began, pre-washed mycelium; tissue was homogenized immediately after the addition of the appropriate lysis buffer (Table 2).

**Table 2:** Composition of modified CTAB (cetyl-trimethyl-ammonium bromide) lysis buffer, with stock concentration and volume details for each protocol. The 3% CTAB + SDS protocol was modified to include 100 ml of Tris(hydroxymethyl)aminomethane and along with the sodium dodecyl sulfate (SDS). The 3% CTAB + PVP included 10 g of Polyvinylpyrrolidone (PVP). We also considered a 2% CTAB extraction along with SDS.

Reagents	3% CTAB + SDS	3% CTAB + PVP	2% CTAB + SDS
Tris-HCl pH 8.0 (1 M)	100 mL	100 mL	100 mL
CTAB (powder)	30 g	30 g	20 g
SDS (10%)	—	—	10 mL/ 10 g
NaCl (5 M/ powder)	280 mL/ 81.9 g	280 mL/ 81.9 g	280 mL/ 81.9 g
EDTA* (0.5 M)	40 mL	40 mL	40 mL
PVP-10 (powder)	—	10 g	—
dH₂O	to 1 L	to 1 L	to 1 L

*EDTA, Ethylenediaminetetraacetic acid.

We compared the extracted DNA in two ways: DNA yield was quantified using the Quantus™ fluorometer, and purity was measured as the 260/280 absorbance ratio via Nanodrop. To evaluate differences in DNA yield as measured by DNA concentration (ng/µL), we fit a linear model where extraction protocol and media type were treated as categorical variables and estimated marginal means for each protocol using the emmeans package [29]. We also extracted estimated marginal means within each media type (Protocol | Media_type) to visualize potential media-specific effects. We fit a similar model for the purity ratio (260/280, Nanodrop) estimates, considering extraction protocol and growth media types as categorical predictors. Although ideal ratios for 260/280 ratios cluster around 1.8, interpretation focused on consistency and relative deviation across groups. Estimated marginal means and 95% confidence intervals were again extracted by protocol and stratified by media type. For both regressions, PD broth was excluded from visualization due to its disproportionately low yield that compressed the plotting scale and obscured meaningful differences among the remaining treatments (S1 Fig). To evaluate how media type influenced DNA concentration and purity, we also fit interaction models for each outcome (DNA concentration and 260/280 ratio), including protocol, media, and their interaction as predictors. The culturing protocol with the fastest growth rate and DNA extraction method with the highest yield described in this peer-reviewed article is published on protocols.io (https://dx.doi.org/10.17504/protocols.io.e6nvw46x9lmk/v1) and is included for printing as S1 File with this article.

Finally, to validate our culturing and extraction protocols, we PCR-amplified the fungal internal transcribed spacer (ITS) barcode region (~500 bp) using the universal primers ITS1 and ITS4 [30], followed by Sanger sequencing. Sequence chromatograms were quality-trimmed, and resulting sequences were queried against both the UNITE and NCBI GenBank databases using BLASTn (v2.15.0+) for taxonomic identification of the fungal isolates associated with BSNB.

## Results

**Fig 2:**
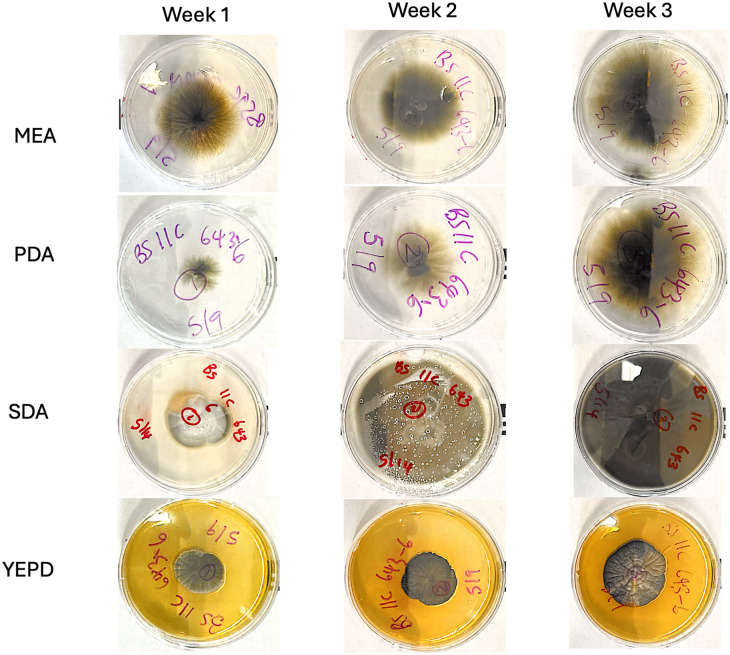
Growth of BSNB associated pathogen on four media malt extract agar (MEA), potato dextrose agar (PDA), sabouraud dextrose agar (SDA)and yeast extract peptone dextrose agar (YEPD) over three weeks. Growth was observed on all four media, with a visual assessment suggesting that sabouraud dextrose agar medium had the most and fastest growth through the third week.

**Fig 3:**
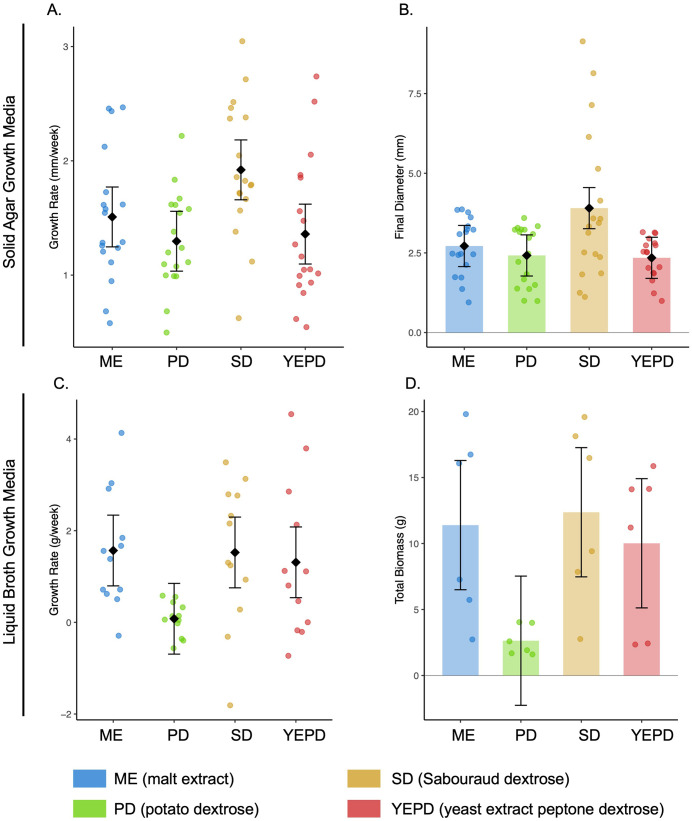
Fungal growth performance across four solid and liquid media types. **(A)** Growth rate (mm/week) and **(B)** final colony diameter (mm) after three weeks on solid media. **(C)** Growth rate (g/week) and **(D)** total biomass (g) after three weeks in liquid culture. Points represent individual replicates; black diamonds indicate estimated marginal means with 95% confidence intervals from linear models. Sabouraud dextrose supported the most robust growth across media formats, while PDA exhibited the lowest performance in both solid and liquid conditions.

We compared fungal growth across four solid media types over a three-week period using six isolates, each tested in three replicates (totaling 72 samples for broth and 72 for solid media). The isolates represent fungal cultures obtained from pine needles showing symptoms of BSNB. All solid media supported visible growth (Fig 2), but SD supported the largest colonies, with an average mean diameter of 3.90 mm whereas ME (2.72 mm), PD (2.42 mm), and YEPD (2.35 mm) supported more limited expansion. When growth was quantified as the weekly change in colony diameter, SD again showed the highest rate (3.34 mm/week), compared to ME (0.83 mm/week), PD (0.69 mm/week), and YEPD (0.31 mm/week). These trends were supported by a linear model of growth rate, which indicated a strong effect of media type (R² = 0.89), with all media types differing significantly from zero growth (all p < 0.001; Fig 3). We also assessed final colony size at the end of the experiment, and SD again yielded the largest diameters (3.91 ± 0.32 mm), with lower values for ME (2.72 ± 0.32), PD (2.42 ± 0.32), and YEPD (2.35 ± 0.32). The linear model for final diameter showed a strong effect of media type (F_4,68_ = 81.15, p < 2.2 × 10 ⁻^16^, adj. R^2^ = 0.82), supporting the conclusion that SD consistently facilitates more extensive fungal growth.

We evaluated fungal growth across four liquid media by measuring weekly weight gain and final biomass after three weeks of incubation. SD broth supported the highest performance, with 74.2 g total biomass, a mean final weight of 12.4 g per replicate, weekly gain of 4.12 g, and a growth rate of 2.14 g/week. ME broth showed comparable outcomes (68.4 g total, 11.4 g mean, 3.80 g/week gain, 1.94 g/week rate), followed by YEPD broth (60.1 g total, 10.0 g mean, 3.34 g/week gain, 1.72 g/week rate). PD broth consistently supported the lowest growth, with just 15.8 g total biomass, a mean of 2.63 g, weekly gain of 0.88 g, and a growth rate of 0.52 g/week. Linear modeling of weekly growth rate revealed significant differences among media (F_4,44_ = 11.08, p = 2.64 × 10 ⁻^6^; Fig 3), with the highest rates observed for ME broth (1.57 ± 0.38 g/week), SD broth (1.52 ± 0.38), and YEPD broth (1.31 ± 0.38), while PD broth exhibited minimal growth (0.08 ± 0.38; Fig 3). A separate model assessing total biomass at week three supported similar trends (F_4,20_ = 17.7, p = 2.37 × 10 ⁻^6^; Fig 3), with significantly higher biomass in SD broth (12.37 ± 2.35 g), ME broth (11.39 ± 2.35), and YEPD broth (10.02 ± 2.35) than in PD broth (2.64 ± 2.35).

**Fig 4:**
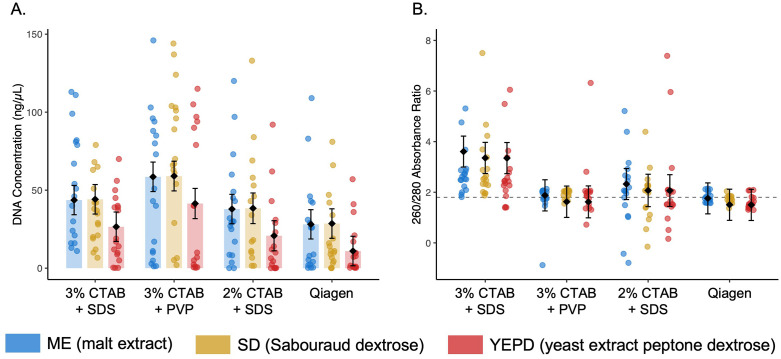
(A) DNA concentration (ng/µL) measured with the Quantus fluorometer varied across extraction protocols and media types. The 2% CTAB + SDS (cetyltrimethylammonium bromide and sodium dodecyl sulfate), 3% CTAB + PVP (polyvinylpyrrolidone), and 3% CTAB + SDS DNA extraction protocols generally yielded higher concentrations than the Qiagen kit. Across all protocols, yields were influenced by media type, with samples grown in potato dextrose (not shown) excluded from final analysis due to its suppressive effect on DNA yield. **(B)** DNA purity assessed by 260/280, where dashed line at 1.8 indicates the conventional target value for pure double stranded DNA. The 2% CTAB + SDS consistently produced values above 1.8, with other protocols closer to this 1.8 benchmark. Bars represent estimated marginal means with 95% confidence intervals from linear models; overlaid points represent individual samples, color-coded by growth media type.

We compared 256 DNA yields across four extraction protocols using Quantus fluorometric quantification and nanodrop, with results analyzed via a linear model that accounted for growth media type. Raw concentration estimates varied substantially among protocols, with 3% CTAB + PVP producing highest average yield (47.3 ± 5.80 ng/µL), followed by 2% CTAB + SDS (31.8 ± 3.62 ng/ µL), and 3% CTAB + SDS (26.6 ± 4.05 ng/ µL), and Qiagen (16.2 ± 2.86 ng/ µL; n = 61−66 per protocol). The linear model confirmed that DNA yield differed significantly across extraction methods and media types (F_7,249_ = 49.57, p < 2.2 × 10 ⁻^16^; adj. R^2^ = 0.565; Fig 4). Estimated marginal means indicated that 3% CTAB +PVP (58.5 ± 4.8 ng/ µL) significantly outperformed all other protocols, while Qiagen produced the lowest yield (28.1 ± 4.8 ng/ µL). Media types also had a strong influence, with solid PD growth media associated with a large negative effect on yield (−40.3 ± 5.7 ng/ µL) and YEPD agar also reducing recovery (−17.1 ± 5.0 ng/ µL) relative to ME agar.

We assessed DNA purity using 260/280 absorbance ratios, where values near 1.8 were considered optimal for pure double-stranded DNA. A linear model including both extraction protocol and media type showed significant differences in purity outcomes (F_7,249_ = 60.86, p < 2.2 × 10 ⁻^16^; adj. R² = 0.621), with extraction protocol exerting the strongest influence on outcomes. Estimated marginal means indicated that Qiagen (1.76 ± 0.31) and 3% CTAB + PVP (1.88 ± 0.31) yielded values closest to the expected purity range, suggesting minimal contamination (Fig 4). The 3% CTAB + SDS produced moderately elevated ratios (2.32 ± 0.31) and 2% CTAB + SDS yielded substantially inflated values (3.61 ± 0.31), suggesting the presence of RNA or other UV-absorbing contaminants. These elevated values were consistent across all media types, with purity estimates for 2% CTAB + SDS ranging from 3.35 to 4.29 (Fig 4).

Furthermore, we assessed whether the effect of extraction protocol on DNA yield and purity depend on medium by fitting a linear model with a protocol and media type interaction. The interaction was significant for DNA concentration (F_15,240_ = 7.10, p < 8.11 × 10 ⁻^13^; adj. R² = 0.2642), and for DNA quality (F_15,240_ = 6.153, p < 6.582 × 10 ⁻^11^; adj. R² = 0.2326), indicating that media modify protocol performance. For example, 3% CTAB + PVP consistently yielded the highest DNA concentrations when isolates were grown on Sabouraud dextrose or malt extract, whereas differences among protocols were smaller when biomass originated from potato dextrose. Similarly, purity differences between protocols were more pronounced for some media than others, reflecting media-specific influences on protocol performance.

Finally, the sequencing of the ITS region resulted in a mean sequence length of 541.3 bp, consistent with the expected amplicon size for fungal ITS regions. The sequencing had an overall mean quality score of 40.2 and a range of 12–57.9, indicating reads suitable for BLAST-based species identification. From the BLASTn (v2.15.0+) searches on UNITE and NCBI databases, the top hits included *Alternaria spp*., *Cladosporium spp*., *Sydowia spp*., *Curvularia spp*., and *Lecanosticta aciola* with percent identity values ranging from 83.85–100% (S2, S3 Files). All samples belonged to Dothideomycetes lineages (S4 File).

## Discussion

Our study identified clear differences in fungal growth and DNA extraction performance across media types and extraction protocols. SD consistently supported the most robust fungal growth, outperforming all other media in both solid agar and liquid broth preparations. Among the DNA extraction methods tested, the 3% CTAB + PVP protocol produced the highest DNA concentrations, while both 3% CTAB + PVP and the Qiagen kit achieved absorbance ratios closest to the 1.8 benchmark for pure double-stranded DNA. In contrast, 3% CTAB + SDS produced elevated 260/280 ratios across all media types, suggesting co-extraction of RNA or other UV-absorbing contaminants that may limit downstream utility. These results collectively point to SD as the most effective medium for culturing BSNB-associated fungi and highlight 3% CTAB + PVP as the most effective extraction strategy for maximizing yield without compromising purity.

The superior performance of Sabouraud dextrose in our assays likely reflects its design as a selective medium for dermatophytes and related filamentous fungi [31]. Sabouraud dextrose agar and broth contains a high dextrose concentration combined with a modest peptone content (Table 1) at an acidic pH (~5.6), conditions that favor fungal metabolism while suppressing bacterial growth [32]. This nutrient and pH profile appears well-suited to fungal taxa in Dothideomycetes, including the BSNB-associated fungi, enabling consistent and rapid colony expansion across both agar and broth contexts. In contrast, PD composed primarily of potato infusion and 2% dextrose offered a less acidic environment and lacked peptides, which may have limited its efficacy for these fungi. Previous studies comparing SD and PD have reported modest but consistent advantages for SD in cultivating clinical fungi, particularly when media selectivity and purity are priorities [33].

The observed superiority of the 3% CTAB + PVP extraction protocol for fungal DNA yield aligned with established biochemical strategies for isolating nucleic acids from plants and fungi rich in polyphenols and polysaccharides [34–36]. PVP acts as a polyphenol-binding agent, reducing oxidative interference and promoting more effective DNA precipitation, an effect documented in CTAB/PVP buffers used for woody species and other fungi [37]. The contrast between 3% CTAB + PVP and 3% CTAB + SDS highlights this distinction: while CTAB+SDS lyses robust fungal cell walls and generates moderate DNA yields, it lacks PVPs phenolic scavenging capacity, likely resulting in inflated 260/280 readings indicative of contamination by RNA or UV-absorbing cellular debris [38,39]. Although the Qiagen kit yielded DNA with near-ideal purity ratios, there was significantly lower DNA concentrations, a limitation consistent with its silica-column mechanism that may bind DNA less efficiently at high input loads [38]. These findings suggest that combining high-salt (3%) precipitation and PVP cleanup maximizes yield from fungal tissue, particularly when a large quantity of DNA is required.

Our findings indicated that while both media type and extraction protocol influence DNA yield and purity, protocol choice exerts the stronger effect on DNA purity, with media acting as a significant modifying factor. Protocols differed consistently in their ability to produce DNA with 260/280 ratios near the expected range for pure double-stranded DNA [20], whereas media effects primarily altered the magnitude of these differences rather than their overall direction. For example, the 3% CTAB + PVP protocol consistently generated the highest yields across all media, but yields were greatest for biomass grown in Sabouraud dextrose and lowest for potato dextrose, mirroring media-specific differences in fungal growth. Similarly, media influenced purity indirectly by affecting fungal biomass composition and potential carryover of media components, which can co-extract with nucleic acids [33]. These results suggest that optimal DNA extraction depends not only on the protocol itself but also on the culturing conditions, with media affecting DNA quality through both biological (e.g., growth rate, metabolite production) and chemical (e.g., carryover) mechanisms [31,32].

While our study identified effective culturing and DNA extraction strategies for BSNB-associated fungi, several limitations temper the generalizability of these findings. First, our assessments were limited to fungi isolated only from symptomatic loblolly pine (*Pinus taeda*) and longleaf pine (*Pinus palustric*) needles; broader validation across a diverse panel of fungal taxa and those associated with other *Pinus* hosts particularly those with varied cell wall compositions or secondary metabolite profiles, would be necessary to extend these recommendations. Finally, while we inferred purity from absorbance ratios, complementary methods such as metabolite profiling, electrophoresis, or high-throughput sequencing could more rigorously quantify the extent and impact of contaminant carryover, especially for protocols with inflated purity values.

Based on our results, we recommend Sabouraud dextrose broth form for culturing BSNB-associated fungi when maximizing DNA yield is the primary goal. This medium reliably provided sufficient mycelial biomass for DNA extraction and other downstream analyses. It consistently supported robust growth across multiple metrics, thereby facilitating greater DNA recovery. For DNA isolation, the 3% CTAB + PVP protocol demonstrated the best performance in terms of both yield and purity, making it the most versatile choice. Our findings confirm that while media type influences fungal growth rate and biomass accumulation, the isolation methods is critical for maintaining DNA integrity and purity. Consistent with our protocol development, we observed that broth cultures generally yielded higher-quality DNA than solid media, largely due to reduced interference from culture components. However, experiment-specific priorities (e.g., DNA yield vs. DNA purity) should guide protocol selection as we identified tradeoffs among alternative extraction approaches that may be favorable for some downstream processes. Isolates used in this study represent diverse fungal species within the class Dothideomycetes class [40], including both pathogenic and endophytes taxa. Endophytic genera such as *Sydowia spp*. [5] and *Clasporium spp* found as secondary invader leaf lesion caused by plant pathogenic fungi [41], were isolated from pinus needles alongside *Lecanosticta acicola* [1]. These results showed that our standardized procedures can streamline research and improve data comparability across fungal pathogen studies, a taxonomic group for which standardized protocols remain lacking despite the ecological and economic effects of fungal infections. By benchmarking the performance of widely available media and DNA extraction approaches, our work addresses a common bottleneck in fungal molecular workflows: achieving high-quality DNA from slow-growing fungi in forest systems. Media type primarily influences fungal growth rate needed for morphological characterization, which is important in plant pathology studies for identifying emerging pathogens. Whereas, DNA isolation protocol has a greater impact on DNA purity and yield, which is critical for molecular and genomic analyses. These findings offer a practical template not only for BSNB research but for broader applications in fungal pathology, where biomass production and nucleic acid purity often constrain downstream genetic and genomic analyses.

## Supporting information

S1 Fig (A) DNA concentration (ng/µL) measured with the Quantus fluorometer varied across extraction protocols and media types. The 2% CTAB + SDS (cetyltrimethylammonium bromide and sodium dodecyl sulfate), 3% CTAB + PVP (polyvinylpyrrolidone), and 3% CTAB + SDS DNA extraction protocols generally yielded higher concentrations than the Qiagen kit. Across all protocols, yields were influenced by media type, with samples grown in potato dextrose supporting particularly low DNA yield. (B) DNA purity assessed by 260/280, where dashed line at 1.8 indicates the conventional target value for pure double stranded DNA. The 2% CTAB + SDS consistently produced values above 1.8, with other protocols closer to this 1.8 benchmark. Bars represent estimated marginal means with 95% confidence intervals from linear models; overlaid points represent individual samples, color-coded by growth media type.
(TIF)

S1 File Step-by-step published protocol pdf version on protocol.io. (PDF)

S2 File Results of the ITS sequence BLAST query for fungal isolates used in this study. Each entry includes the query ID, GenBank accession number, length, percent identity, e-value, bit score, and the corresponding species identified from NCBI database.
(CSV)

S3 File Results of the ITS sequence query against the UNITE fungal database. Each record includes the reference ID, taxon name, similarity threshold (SH), alignment score, e-value, and percent identity, providing a summary of species-level identifications for the isolates analyzed in this study.
(CSV)

S4 File FASTA formatted sequences of isolates used in this study. (CSV)
