# Supplementary figures and images for: Optimized protocol for culturing and extracting DNA from fungal isolates associated with brown spot needle blight in pine trees
Source: PLoS One. 2025 Nov 19;20(11):e0337218. doi: 10.1371/journal.pone.0337218 (PMC12629444; doi:10.1371/journal.pone.0337218)

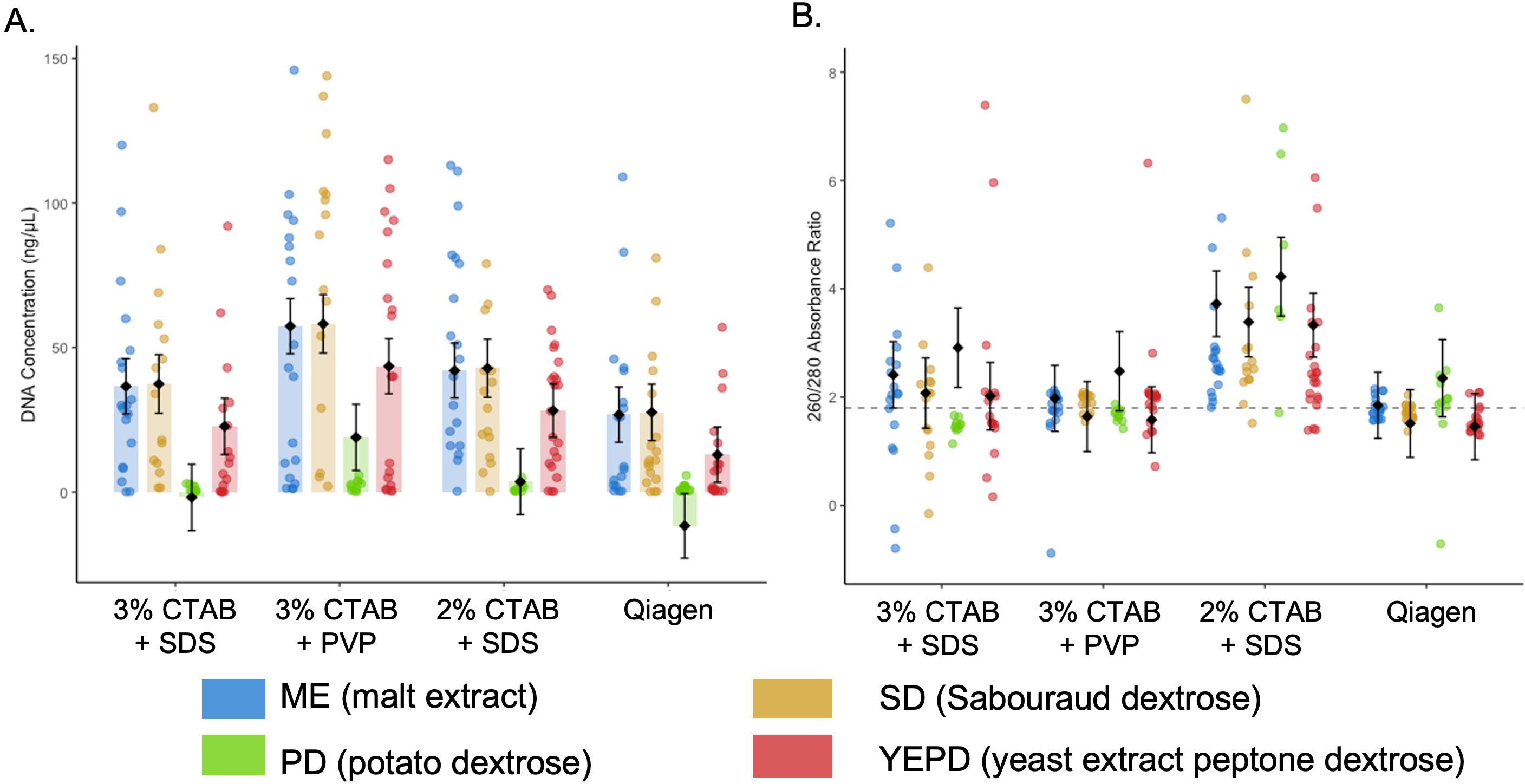

Supplement: S1 Fig — The 2% CTAB + SDS (cetyltrimethylammonium bromide and sodium dodecyl sulfate), 3% CTAB + PVP (polyvinylpyrrolidone), and 3% CTAB + SDS DNA extraction protocols generally yielded higher concentrations than the Qiagen kit. Across all protocols, yields were influenced by media type, with samples grown in potato dextrose supporting particularly low DNA yield. (B) DNA purity assessed by 260/280, where dashed line at 1.8 indicates the conventional target value for pure double stranded DNA. The 2% CTAB + SDS consistently produced values above 1.8, with other protocols closer to this 1.8 benchmark. Bars represent estimated marginal means with 95% confidence intervals from linear models; overlaid points represent individual samples, color-coded by growth media type. (TIF) [file pone.0337218.s001.tif]
