# Supplementary material for: Optimized protocol for culturing and extracting DNA from fungal isolates associated with brown spot needle blight in pine trees
Source: PLoS One. 2025 Nov 19;20(11):e0337218. doi: 10.1371/journal.pone.0337218 (PMC12629444; doi:10.1371/journal.pone.0337218)
Supplement: S1 File — (PDF) [file pone.0337218.s002.pdf]

Oct 14, 2025

Protocol for culturing and extracting DNA from fungal isolates associated with brown spot needle blight

DOI
dx.doi.org/10.17504/protocols.io.e6nvw46×9lmk/v1

Temitope R. Folorunso[1], Gabriel Silva[1], Marilis E. Girón[2], Tess Lindow[1], Micah Persyn[1], Lori Eckhardt[1], Janna R. Willoughby[1]

[1]College of Forestry, Wildlife, and Environment, Auburn University, Auburn, Alabama.;
[2]Department of Agricultural Sciences, Zamorano University, Municipio de San Antonio de Oriente, Francisco Morazán, Honduras, 11101 Tegucigalpa, Honduras

Temitope Folorunso
Auburn University

Create & collaborate more with a free account

Edit and publish protocols, collaborate in communities, share insights through comments, and track progress with run records.

Create free account

OPEN ACCESS

DOI: https://dx.doi.org/10.17504/protocols.io.e6nvw46x9lmk/v1

Protocol Citation: Temitope R. Folorunso, Gabriel Silva, Marilis E. Girón, Tess Lindow, Micah Persyn, Lori Eckhardt, Janna R. Willoughby 2025. Protocol for culturing and extracting DNA from fungal isolates associated with brown spot needle blight. **protocols.io https://dx.doi.org/10.17504/protocols.io.e6nvw46x9lmk/v1**

License: This is an open access protocol distributed under the terms of the **Creative Commons Attribution License**, which permits unrestricted use, distribution, and reproduction in any medium, provided the original author and source are credited

Protocol status: Working
We use this protocol and it's working

Created: June 13, 2025

Last Modified: October 14, 2025

Protocol Integer ID: 222314

Keywords: Media, Fungal isolates, Cetyltrimethylammonium bromide, Pathogen , Brown spot needle blight, fungal pathogen dna from pine needle, brown spot needle blight isolation of fungal pathogen, dna from fungal isolate, isolating fungal pathogen dna, fungal isolate, understanding fungal pathogen, fungal pathogen, modified cetyltrimethylammonium bromide dna extraction protocol, cetyltrimethylammonium bromide dna extraction protocol, brown spot needle blight isolation, dna extraction, extracting dna, brown spot needle blight, efficient dna extraction protocol, greatest fungal growth, pathogen, pine needle, yeast, extraction, dna

Funders Acknowledgements:
USFS
Grant ID: G00015817, LGE and JRW

Abstract

Isolation of fungal pathogens and efficient DNA extraction protocols are critical steps for enhancing downstream molecular applications. In this protocol, we demonstrate an effective approach for isolating fungal pathogen DNA from pine needles with brown spot needle blight (BSNB) symptoms. We used solid malt extract agar and Sabouraud dextrose broth, which supported the greatest fungal growth over a three-week period in our tests comparing these to potato dextrose agar and yeast extract peptone dextrose. We then used a modified cetyltrimethylammonium bromide DNA extraction protocol to extract high molecular weight DNA. These methods will support future research efforts aimed at understanding fungal pathogens that infect pine needles.

Materials

Sorbitol wash buffer:

	A	B	C	D
		Final concentration	Stock	For 1L
	Tris-HCl pH 8.0	100 mM	1 M	100 mL
	Sorbitol	0.35 M	Powder	63.76 g
	EDTA	5 mM	0.5 M	10 mL
	PVP-40	1%	Powder	10 g

CTAB buffer:

	A	B	C	D
		Final concentration	Stock concentration	1L
	Tris-HCl pH 8.0	100 mM	1 M	100 mL
	CTAB	3%	Powder	30 g
	NaCl	1.4 M	Powder / 5 M	81.9 g / 280 mL
	EDTA	20 mM	0.5 M	40 mL
	PVP-40	10 g/L	Powder	10 g

Troubleshooting

Isolate Culturing

5d 1h 22m

1

Note

All reagents were commercially purchased, mixed and modified according to existing protocol

Preparation of 1 L of solid 3 % Malt extract agar (MEA) medium

1.1 Weigh 30 g of malt extract.

1.2 Weigh 15 g of DifcoTM Bacto Agar.

1.3 Weigh peptone of 5 g (Er et al., 2015).

1.4 Measure 1000 mL of distilled water.

1.5 Mix all in Erlenmeyer flask 1000 mL (PYREX).

1.6 Add a magnetic stirrer and put on Fisher Thermix Stiring hotplate at 60 °C .

1.7 Allow mixture to be cleared and free of particles.

1.8 Autoclave for 00:20:00 at 121 °C . 20m

1.9 Allow media to cool briefly.

1.10 Clean the Flow hood with conflicts followed by 70% ethanol.

1.11 Expose the flow hood to UV light for 00:10:00 . 10m

1.12 Use sterilized packs of petri dishes 100 ×15 mm (VWR).

1.13 Pour ~ 30 mL media into the petri dishes under laminar flow hood.

1.14 Allow medium to circulate the bottom of plate.

1.15 Incubate for ~ 48:00:00 at 22 °C to confirm no contamination. 2d

2 Preparation of 1 L of solid Sabouraud dextrose agar (SDA) medium

2.1 Weigh 40 g of dextrose.

2.2 Weigh 10 g of peptone.

2.3 Weigh 15 g of DifcoTM Bacto Agar.

2.4 Measure 1000 mL of distilled water.

2.5 Mix all in Erlenmeyer flask 1000 mL (PYREX).

2.6 Add a magnetic stirrer and put on Fisher Thermix Stiring hotplate at 60 °C .

2.7 Allow mixture to be cleared and free of particles.

2.8 Autoclave for 00:20:00 at 121 °C . 20m

2.9 Allow media to cool briefly.

2.10 Clean the flow hood with conflicts followed by 70% ethanol.

2.11 Expose the flow hood to UV light for 00:10:00 . 10m

2.12 Use sterilized packs of petri dishes 100 ×15 mm (VWR).

2.13 Pour ~ 30 mL media into the petri dishes under laminar flow hood.

2.14 Allow media to circulate the bottom of plate.

2.15 Incubate for ~ 48:00:00 at 22 °C to confirm no contamination. 2d

3 **Preparation of 1 L Sabouraud dextrose (SD) broth**

3.1 Weigh 40 g of dextrose.

3.2 Weigh 10 g of peptone.

3.3 Measure 1000 mL of distilled water.

3.4 Mix all in Erlenmeyer flask 1000 mL (PYREX).

3.5 Add a magnetic stirrer and put on Fisher Thermix Stiring hotplate at 60 °C .

3.6 Allow mixture to be cleared and free of particles.

3.7 Distribute 75 mL into 125 mL Erlenmeyer flask (PYREX).

3.8 Cover with cotton plugs and seal with aluminum foil.

3.9 Autoclave for 00:20:00 at 121 °C . 20m

3.10 Allow to sit for ~ 24:00:00 . 1d

4 Needle surface sterilization and fungal culturing

4.1 Process symptomatic needles within three days of field collection.

4.2 Remove 4-5 needles strands from samples.

4.3 Cut needles into 3-4 cm pieces.

4.4 Pore inside a beaker and guide by white cheese clothes.

4.5 Dip the clothes containing needles in 7.5% sodium hypochlorite for 00:00:30 . 30s

4.6 Rinse with distilled water for 00:00:30 . 30s

4.7 Wash again with 70% ethanol for 00:00:30 (Barnes et al 2014). 30s

4.8 Rinse with distilled water for 00:00:30 . 30s

4.9 Place the cloth with needles aside and allow to dry.

4.10 In an aseptic environment, place the cleaned needles on the petri dishes with media.

4.11 Give it a radial pattern arrangement.

4.12 Prepare each sample in four replicates.

4.13 Seal plate with parafilm.

4.14 Arrange plates in a tray and allow it to grow at 37 °C .

4.15 Check plates for sporulation and growth weekly.

5 **Subculturing for pure isolate**

5.1 Identify distinct colonies grown around the needles from the previous step.

5.2 Scrape the edge of each colony into a small media plate (petri dishes 60mm).

5.3 Seal plate with parafilm.

5.4 Make triplicate of samples per colony.

6 **Inoculation of pure fungal on liquid broth**

6.1 Scrape tip of the pure fungal colony using forceps.

6.2 Inoculate pure fungal culture on liquid broth in the 125 mL Erlenmeyer flasks.

6.3 Seal with cotton plug and aluminum foil.

6.4 Arrange in a tray.

6.5 Set the Room temperature to 37 °C .

6.6 Label sample accordingly.

DNA Extraction

12h 55m

7

Note

All reagents were commercially purchased, mixed and modified according to existing protocol.

Pre-washing of Mycelia

7.1 Harvest mycelia after one to two weeks of incubation in media broth.

7.2 Transfer the mycelia to sterilized 50 mL falcon tubes.

7.3 Centrifuge at 5000 x g, 00:05:00 with Thermoscientifc multifuge X pro series. 5m

7.4 Remove supernatant media.

7.5 Add 1 mL of 1X phosphate-buffered saline (PBS, pH 7.5) .

7.6 Mix vigorously by vortexing.

7.7 Centrifuge at 5000 x g, 00:05:00 . 5m

7.8 Repeat wash until clean mycelial pellets is obtained, effectively removing media residues.

8 **Prepare sorbitol wash buffer (Inglis et al., 2018)**

	A	B	C	D
		**Final concentration**	**Stock**	**For 1L**
	Tris-HCl pH 8.0	100 mM	1 M	100 mL
	Sorbitol	0.35 M	Powder	63.76 g
	EDTA	5 mM	0.5 M	10 mL
	PVP-40	1%	Powder	10 g

8.1 Weigh and measure all reagents according to desired final volume.

8.2 Mix all reagent in Erlenmeyer flask 1000mL (PYREX) with magnetic stirrer.

8.3 Add magnetic stirrer and place on Fisher Thermix Stiring hotplate on 60 °C .

8.4 Allow the mixture to be thoroughly dissolved without any particles.

8.5 Autoclave for 00:20:00 at 121 °C . 20m

8.6 Immediately before use, mix 10 µL of β-Mercaptoethanol (β-ME) (Lot no 23F2056260) to each mL of sorbitol buffer (1% v/v), now referred as SWB-βME.

8.7 Weigh Approximately 200 mg - 300 mg of fungal mycelia.

8.8 Transfer into a 2 mL screw-cap tube.

8.9 Add 1 mL of the prepared SWB-βME solution.

Note

Prepare mastermix for easy pipetting.

8.10 Add a beating bead to each sample.

8.11 Disrupt the cell using a precellys at 9100 RPM for three cycles of 00:00:30 each, with 5-second pauses between each cycle.

8.12 Centrifuge homogenized mixtures at 17000 x g, 00:05:00 . 5m

8.13 Discard the supernatant.

8.14 Repeat the process until it is clean, particularly for sticky mycelial pellets that failed to settle.

9 **Prepare 3% CTAB buffer (Schenk et al., 2023)**

	A	B	C	D
		**Final concentration**	**Stock concentration**	**1L**

	A	B	C	D
	Tris-HCl pH 8.0	100 mM	1 M	100 mL
	CTAB	3%	Powder	30 g
	NaCl	1.4 M	Powder / 5 M	81.9 g / 280 mL
	EDTA	20 mM	0.5 M	40 mL
	PVP-40	10 g/L	Powder	10 g

9.1 Weigh and measure all reagents according to desired final volume, freshly prepared 10 g Polyvinylpyrrolidone (PVP-40) is advisable.

9.2 Mix all reagent in Erlenmeyer flask 1000mL with magnetic stirrer.

9.3 Add a magnetic stirrer and put on Fisher Thermix Stiring hotplate on 60 °C .

9.4 Allow the mixture to be thoroughly dissolved.

9.5 Autoclave for 00:20:00 at 121 °C . 20m

9.6 Immediately before extraction, add PVP-40.

10 **CTAB extraction**

10.1 Add 1 mL of 3% CTAB buffer to sorbitol washed mycelia.

10.2 Add 15 µL of β-ME.

10.3 Add 15 µL of proteinase K (20 µg/µl).

10.4 Disrupt the cell again using a precellys at 9100 RPM for three cycles of 00:00:30 each, with 5-second pauses between each cycle.

> **Note**
>
> Prepare mastermix for easy pipetting.

10.5 Incubate mixture for 03:00:00 on a Incubating microplate shaker (VWR) at 65 °C . 3h

10.6 Rigorously shake the mixture again using precellys.

10.7 Centrifuge at 17000 x g, Room temperature, 00:10:00 . 10m

10.8 Transfer the supernatant into a 2ul new tube.

10.9 Add equal volume of phenol-chloroform-isoamyl alcohol (25:24:1).

10.10 Invert the mixture ~50.

10.11 Incubate at Room temperature for 00:05:00 . 5m

10.12 Centrifuge at 17000 x g, 00:10:00 . 10m

10.13 Transfer upper phase to a fresh 2ul tube.

10.14 Add equal volume of chloroform:isoamyl alcohol (24:1).

10.15 Gently invert the mixture ~30.

10.16 Incubate for 00:05:00 . 5m

10.17 Centrifuge at 17000 x g, 00:10:00 . 10m

10.18 Remove the resulting supernatant to a new tube.

10.19 Add 100 µg/µl RNase A (5 µL).

10.20 Incubate at 37 °C for 00:10:00 . 10m

10.21 Add 0.2 volumes of [M] 10 Molarity (M) ammonium acetate (NH_4Ac) and 0.8 volumes of ice-cold 100% isopropanol to RNase treated mixture.

10.22 Invert mixture ~20 times.

10.23 Incubate at -20 °C Overnight , to precipitate the DNA. 8h

10.24 Centrifuge at 17000 x g, 00:10:00 to pellet the DNA. 10m

10.25 Discard the resulting supernatant.

10.26 Wash pellets with 70% ethanol.

10.27 Repeat the wash.

10.28 Remove residual liquid using a pipette.

10.29 Air-dry the mixture.

10.30 Resuspend DNA in nuclease-free water (50 µL - 100 µL).

10.31 Store at 4 °C .

Note

Notes:
1. All prepared buffers can be stored in 20°C except CTAB.
2. β-Mercaptoethanol should be stored in flammable cabinet

Protocol references

References:

1. Er, C. M., Sunar, N. M., Leman, A. M., & Othman, N. (2015). Direct growth inhibition assay of total airborne fungi with application of biocide-treated malt extract agar. MethodsX, 2, 340–344. https://doi.org/10.1016/j.mex.2015.07.002

2. Inglis, P. W., Pappas, M. de C. R., Resende, L. V., & Grattapaglia, D. (2018). Fast and inexpensive protocols for consistent extraction of high quality DNA and RNA from challenging plant and fungal samples for high-throughput SNP genotyping and sequencing applications. PLOS ONE, 13(10), e0206085. https://doi.org/10.1371/journal.pone.0206085

3. Schenk, J. J., Becklund, L. E., Carey, S. J., & Fabre, P. P. (2023). What is the “modified” CTAB protocol? Characterizing modifications to the CTAB DNA extraction protocol. Applications in Plant Sciences, 11(3), e11517.
